# Maternal immune activation results in complex microglial transcriptome signature in the adult offspring that is reversed by minocycline treatment

**DOI:** 10.1038/tp.2017.80

**Published:** 2017-05-09

**Authors:** D Mattei, A Ivanov, C Ferrai, P Jordan, D Guneykaya, A Buonfiglioli, W Schaafsma, P Przanowski, W Deuther-Conrad, P Brust, S Hesse, M Patt, O Sabri, T L Ross, B J L Eggen, E W G M Boddeke, B Kaminska, D Beule, A Pombo, H Kettenmann, S A Wolf

**Affiliations:** 1 Cellular Neurocience, Max-Delbrueck-Center for Molecular Medicine in the Helmholtz Association, Berlin, Germany; 2 Core Unit Bioinformatics, Berlin Institute of Health, Berlin, Germany; 3 Charite Medical University, Berlin, Germany; 4 Epigenetic Regulation and Chromatin Architecture Group, Berlin Institute for Medical Systems Biology, Max-Delbrück Centre for Molecular Medicine in the Helmholtz Association, Berlin, Germany; 5 Institute of Cell Biology and Neurobiology, Charité-Universitaetsmedizin, Berlin, Germany; 6 Department of Neuroscience, Section Medical Physiology, University Medical Center Groningen, University of Groningen, Groningen, The Netherlands; 7 Laboratory of Molecular Neurobiology, Nencki Institute of Experimental Biology, Warsaw, Poland; 8 Department of Neuroradiopharmaceuticals, Institute of Radiopharmaceutical Cancer Research, Research Site Leipzig, Helmholtz-Zentrum Dresden-Rossendorf, Leipzig, Germany; 9 Department of Nuclear Medicine, University of Leipzig, Leipzig, Germany; 10 Integrated Treatment and Research Centre (IFB) Adiposity Diseases, University of Leipzig, Leipzig, Germany; 11 Department of Nuclear Medicine, Hannover Medical School, Hannover, Germany; 12 Max-Delbrueck-Center for Molecular Medicine in the Helmholtz Association, Berlin, Germany

## Abstract

Maternal immune activation (MIA) during pregnancy has been linked to an increased risk of developing psychiatric pathologies in later life. This link may be bridged by a defective microglial phenotype in the offspring induced by MIA, as microglia have key roles in the development and maintenance of neuronal signaling in the central nervous system. The beneficial effects of the immunomodulatory treatment with minocycline on schizophrenic patients are consistent with this hypothesis. Using the MIA mouse model, we found an altered microglial transcriptome and phagocytic function in the adult offspring accompanied by behavioral abnormalities. The changes in microglial phagocytosis on a functional and transcriptional level were similar to those observed in a mouse model of Alzheimer’s disease hinting to a related microglial phenotype in neurodegenerative and psychiatric disorders. Minocycline treatment of adult MIA offspring reverted completely the transcriptional, functional and behavioral deficits, highlighting the potential benefits of therapeutic targeting of microglia in psychiatric disorders.

## Introduction

In recent years, microglia as the brain’s intrinsic immune cells have emerged as having new roles in the pathophysiology of psychiatric disorders such as schizophrenia.^1^ Signs of their involvement stem from human postmortem and positron emission tomography studies indicating altered microglial cell density and binding to the translocator protein (TSPO) in the brains of a subset of schizophrenic patients.^2, 3, 4^ Microglia are able to phagocytose synaptic material in the process of synaptic pruning, which is essential for the proper development and maintenance of brain circuitry.^5^ Their phagocytic activity also controls the pool of neuronal progenitor cells during development and in adulthood.^6, 7^ As schizophrenia is a disorder with prominent abnormalities in synaptogenesis,^8^ neurogenesis^9, 10^ and neuronal transmission,^8, 11^ it is plausible that microglial dysfunction results in aberrant synaptogenesis and neurogenesis in the developing central nervous system, contributing to the development and progression of schizophrenia. Epidemiological studies have linked prenatal influenza infections with an increased risk of developing schizophrenia in the offspring.^12, 13^ Injection of the viral mimic polyinosinic:polycytidilic acid (Poly(I:C)) to pregnant rodent dams has been shown to induce a series of behavioral, morphological, cytoarchitectural and biochemical changes in the brain of adult offspring, which are highly comparable to the abnormalities observed in human schizophrenic patients.^14, 15^ The maternal immune activation (MIA) in humans and animal models alike could impact on the fetal microglia and result in functional changes that are retained in adulthood.

Several clinical case reports have suggested that minocycline alleviates mainly negative symptoms in schizophrenic patients. Subsequent clinical trials were initiated to test the potential of the drug as an add-on therapy for schizophrenia. In spite of the accumulating data showing beneficial effects of minocycline treatment on both human patients^16, 17, 18, 19, 20, 21, 22^ and in animal models of schizophrenia,^23, 24^ a mechanistic insight into the action of minocycline is lacking at present. It is suggested that minocycline interferes with microglial activation; however, its actions may be nonselective to microglial function. Chronic inflammation and, in particular, MIA could be a major factor that contributes to the development of neurodegenerative disorders such as Alzheimer’s disease.^25, 26, 27^ Lahiri *et al.*^28^ proposed a 'Latent Early-life Associated Regulation' model, postulating that latent changes in the expression of specific genes initially primed at the developmental stage of life. However, the involvement of microglia in neuropsychiatric diseases is unclear.

Therefore, using the maternal immune activation model, we investigated the effects of minocycline treatment on the phenotype of hippocampal microglia derived from adult male offspring. We also compared these data sets to the ones obtained from an Alzheimer’s disease mouse model. We focused here on the hippocampus as it is a structure particularly affected in schizophrenia, and its malfunction contributes to the appearance of positive, negative and cognitive symptoms.^29, 30, 31, 32^

## Materials and methods

### Animals

**Figure 1:**
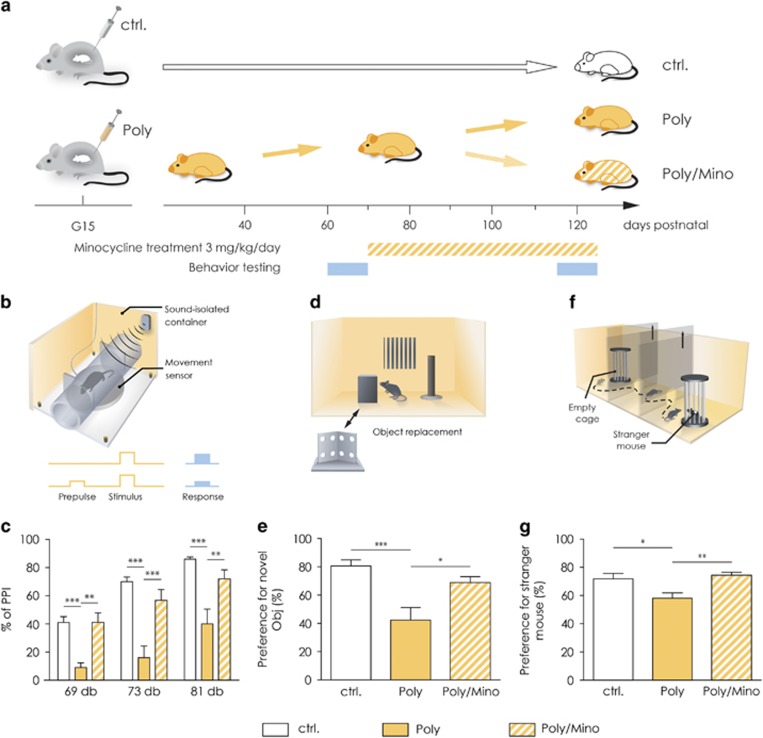
Poly(I:C) mice show correlates of positive, negative and cognitive symptoms, which are rescued by chronic minocycline treatment. (**a**) Representative image showing the experimental design. Pregnant dams were injected at gestational day 15 (G15) with either saline solution or Poly(I:C). The offspring was tested for behavioral deficits at postnatal day 60 (P60). Poly(I:C) animals were then subjected to minocycline treatment (3 mg kg^−1^ per day) for 5 weeks. They were then re-tested for the same behavioral battery at the end of the treatment. (**b**) Representation of the apparatus used for the prepulse inhibition test (PPI). (**c**) Part of the adult offspring of Poly(I:C)-challenged dams exhibited deficits in sensorimotor gating as measured through the PPI test for all three prepulses tested, (69, 73 and 81 dB prepulses). Minocycline treatment significantly restored the percentage of PPI for all three prepulses (*n*=7 Poly(I:C) mice before and after minocycline treatment and 14 controls). (**d**) Representative image of the apparatus used for the novel object recognition test. (**e**) Poly(I:C) mice showed working memory impairments in the novel object recognition task, displayed as a significantly decreased percentage of time spent making contact with a new object as compared with control animals. Five-week minocycline treatment significantly increased their ability to detect a novel object. (*n*=16 Poly(I:C) mice before and after minocycline treatment and 14 controls). (**f**) Representative image of the apparatus used for the sociability test. (**g**) Poly(I:C) mice display deficits in social behavior shown as decreased percentage of time spent making contact with a stranger mouse as compared with controls. Five-week minocycline treatment significantly restored a normal social pattern in these mice (*n*=16 Poly(I:C) mice before and after minocycline treatment and 14 controls). Error bars indicate s.e.m. in all the panels. The PPI data were analyzed by two-way analysis of variance (ANOVA) followed by Bonferroni *post hoc* test, while the novel object recognition test and the sociability data by one-way ANOVA followed by Bonferroni *post hoc* test. *P<0.05, **P<0.001, ***P<0.0001. Ctrl, control animals; dB, decibel; G15, gestational day 15; Obj, object; Poly, Poly(I:C) animals; Poly/Mino, Poly(I:C) animals treated with minocycline; PPI, prepulse inhibition.

The C57BL/6 mice were handled according to governmental (LaGeSo) and internal (MDC) rules and regulations (animal protocol number G O196/12). Day one of pregnancy was calculated from the day of appearance of a vaginal plug. Pregnant dams were injected once intraperitoneally with either Polyinosinic:polycytidylic acid (5 mg kg^−1^ Poly(I:C) VacciGrade, Invivogen, San Diego, CA, USA) or 0.9% saline solution at gestational day 15 (G15, Figure 1a). Despite its advantages, there are also limitations to the Poly(I:C)-based MIA animal paradigm. Apart from the ‘traditional’, general concerns relating to the usage of rodent systems to model complex neuropsychiatric disorders, which present with alterations of inherently ‘human’ behavioral features, there are also experimental constrains of Poly(I:C)-assisted MIA that need to be considered. The major drawback of using immunogenic manipulations based upon Poly(I:C), rather than live pathogens, is the—albeit well-characterized—limited set of immune responses elicited. Comparatively, stimulating the maternal immune system by administration of a real virus (such as influenza), leads to a much broader activation of not only the innate, but also the acquired immune system, thus more closely resembling the situation following infection during pregnancy in humans. To ensure a least of five animals per group for every set of experiments, an average of 10 dams per group (Poly(I:C) and saline injected) was used. Male offspring were weaned at postnatal day 21 (P21) and they were caged in a random manner so as to avoid confounding litter effects. The mice were left undisturbed until the day of behavioral phenotyping with the prepulse inhibition (PPI) test at P60. The latter was used as a selection criteria so that only the Poly(I:C) mice displaying deficits in PPI were selected for further experiments. In Supplementary Figure 3, we show all animals tested with PPI and indicate the ones that were included for further analysis. The mice were kept in an animal facility with 12 h of light and dark cycle with food and water *ad libitum*.

### Minocycline treatment

Minocycline (Sigma-Aldrich, Munich, Germany) was dissolved in drinking water at 3 mg kg^−1^ per day assuming an average consumption of 5 ml water per day per animal. The dose was selected on the basis of the oral dosage given to schizophrenic subjects in clinical trials (200 mg per day assuming 70 kg weight, reviewed in ref. 33). Minocycline treatment was started at postnatal day (P)70–P80 after the assessment of the baseline behavioral deficits, and was carried out for 5 weeks and throughout the behavioral testing period (Figure 1a). On treatment, the mice were randomly divided into the following groups: (Ctr), Controls—offspring from NaCl-treated dams; Poly—offspring of Poly(I:C)-treated dams; and Poly/Mino—offspring from Poly(I:C)-treated dams that received minocycline as adults. We have previously reported that the minocycline treatment given to control animals did not affect the sensorimotor gating, adult hippocampal neurogenesis, microglial cell density and cytokine expression pattern.^23^

### Behavioral testing

Previous work conducted on Poly(I:C) offspring showed that they start to display behavioral deficits at the switch between adolescence and early adulthood, as it is the case in human schizophrenic patients (reviewed in ref. 34). This age corresponds in mice to P60. We therefore started the behavioral battery tests at P60. The behavioral testing was performed by a person blind to the experimental groups.

### Prepulse inhibition

The PPI of startle reflex was assessed using a standard startle chamber (Figure 1b, SR-Lab, San Diego Instruments, San Diego, CA, USA) according to the manufacturer's protocol. The session starts with 5 min habituation where the mouse is exposed to white noise at 65 decibel (dB). Afterwards, a pseudorandomized session started with 10 different types of trials: 8 acoustic startle pulse alone (120 dB) and 10 different prepulse followed by pulse trials were applied in which either 69, 73 or 81 dB stimuli were presented 100 ms before the pulse. Intertrial intervals of a random duration of 10–25 s (65 dB white noise) separated the trials. In the prepulse plus startle pulse trials, the amount of PPI is measured and expressed as percentage of the basal startle response (startle pulse alone).

### Novel object recognition test

The test was performed in a square-shaped open-field box with high walls (72 × 72 × 33 cm, Figure 1d). The test was performed as described elsewhere, but with some modifications.^35^ The test starts with a 5 min habituation session. After an intertrial interval of 5 min, the mouse is allowed to explore two different objects for 5 min. After an intertrial interval of 5 min, the mouse was allowed to explore the same arena for 3 min containing one old and one novel object (Figure 1d). The measurements were performed through the Anymaze software (ANY-maze 4.50, Stoelting Europe, Dublin, Ireland). To assess the detection of novelty, we calculated the percentage of time spent by the mice exploring the novel object (NO) as compared with the total time spent in exploring the new and old object (OB):

(NO_ExplorationTime_/(NO_ExplorationTime_+OB_ExplorationTime_)) × 100. The open field and the objects were cleaned with 5% ethanol after each mouse was tested, to avoid olfactory cues.

### Sociability test

To assess the social behavior of the mice, the sociability test was performed in a tripartite chamber, composed of three chambers of equal size (20 × 40 cm, Figure 1f). After habituation for 5 min in the middle chamber, two cylindrical cages, one empty and one containing a stranger mouse were placed in the lateral chambers. The mouse was given then free access to all chambers for a total of 10 min (ANY-maze 4.50, Stoelting Europe). The preference for the stranger mouse (S) toward the empty cage (E) was assessed by calculating the percentage of time spent making contact with the stranger mouse over the sum of time spent with both the S and the E: (S_TimeMakingContact_/(S_TimeMakingContact_+E_TimeMakingContact_)) × 100. The cages and chambers were wiped clean with 5% ethanol after each mouse to avoid olfactory cues.

### Magnetic cell sorting of microglial cells

The mice were deeply anesthetized with pentobarbital (Narcoren, Merial, Hallbergmoos, Germany) and perfused with ice-cold phosphate-buffered saline. After decapitation, the head was removed and the brain retrieved and put immediately in ice-cold Hank's balanced salt solution (GIBCO, Invitrogen, Karlsruhe, Germany). Tissue dissection and microglial isolation were performed as described elsewhere.^36^ Briefly, the pelleted single-cell suspension of the hippocampi was resuspended in ice-cold percoll solution composed of myelin gradient buffer plus percoll (GE Healthcare, Little Chalfont, Buckinghamshire, UK) and a layer of phosphate-buffered saline was carefully applied on top. The tubes were then centrifuged at 950 g, 4 °C low brake and acceleration. The cells were resuspended and washed in ice-cold magnetic cell sorting (MACS) buffer (phosphate-buffered saline, 0.5% bovine serum albumin, 2 mm EDTA) followed by incubation with anti-mouse CD11b magnetic beads (Miltenyi Biotech, Bergisch Gladbach, Germany) at 4 °C for 15 min. The cells were resuspended in MACS buffer and passed through medium-sized MACS columns (Miltenyi Biotech) attached to a magnet. The flow-through was discarded and the cells were flushed out of the column in MACS buffer, pulled down by centrifugation and resuspended in 1 ml TRIzol (Thermo Scientific, Schwerte, Germany) snap frozen in liquid nitrogen and stored at −80 °C until library preparation.

### mRNA library preparation and sequencing

The messenger RNA (mRNA) libraries were made using TruSeq RNA Sample Preparation Kits v2 setA (#RS-122-2001, Illumina, San Diego, CA, USA) following the manufacturer's instructions. RNA-sequencing (RNA-seq) was performed on mRNA extracted from freshly isolated microglia (as described above) from the hippocampi of control, Poly(I:C) and minocycline-treated Poly(I:C) animals. The Poly(I:C) animals used for the RNA-seq were chosen on the basis of their deficit in PPI compared with control animals (Supplementary Figure 1a). A group of Poly(I:C) animals were treated with minocycline (3 mg kg^−1^ per day) for 5 weeks and re-tested for PPI to confirm the efficacy of minocycline on this schizophrenic endophenotype. A total amount of six animals per group were used. To obtain a substantial number of microglial cells, hippocampi were pooled together from three animals per group for a final number of two biological replicates per group. The complementary DNA (cDNA) libraries were loaded onto a HiSeq Rapid PE Flow Cell (Illumina) and mRNA-seq libraries were sequenced paired-end (2 × 100 bp) using a HiSeq 2500 sequencer (Illumina).

### Quantitative PCR

For quantitative PCR validation of selected genes, we used the RNA extracted for the sequencing experiment. First-strand cDNA synthesis was done with the SuperScript II reverse transcriptase (Invitrogen, Carlsbad, CA, USA) using oligo-dT primers_12__–__18_ (Invitrogen) according to the manufacturer’s instructions. Quantitative real-time PCR reactions were performed in a 7500 Fast Real-Time thermocycler (Applied Biosystems, Carlsbad, CA, USA) using the SYBR Select Master Mix (Applied Biosystems) according to the manufacturer’s instructions. cDNA input ranged between 1 and 5 ng μl^−1^ of total RNA transcribed into cDNA. The expression data were normalized to beta-actin. The primers used are listed in Supplementary Material 1.

### Computational analysis

Paired-end RNA-Seq reads were mapped to the mouse reference genome GCRM38/mm10 with STAR,^37^ using the following parameters ‘—outFilterMultimapNmax 20 —alignSJoverhangMin 8 —alignSJDBoverhangMin 1 –outfilterMismatchNmax 999 —outFilterMismatchNovelLmax 0.04 —alignIntronMin 20 —alignIntronMax 10^6 —alignMatesGapMax 10^6’. Mapped read pairs were assigned to genes with feature Counts.^38^ We used gencode version M6 (Ensembl 81) mouse genome annotation. Alternative scaffolds, fix- and novel- patches were removed from the analysis. To identify differentially expressed genes between different conditions, we used R/Bioconductor DeSeq2.^39^ The analysis was carried out with DeSeq2 default parameters, using raw read counts. Gene ontology (GO) analysis was performed with topGO gene ontology tool, using the ‘elim’ algorithm (nodes=5; Alexa A and Rahnenfuhrer J 2016, topGO: Enrichment Analysis for Gene Ontology, R package version 2.24.0.).

For the comparison of differential expression with APP mouse model, we downloaded the log2 fold changes and the adjusted *P*-values from Holtman *et al.*^40^ This (microarray) data set comprised 9938 genes of which 9813 could be matched with our Gencode M6 gene IDs. Among these, 1595 were differentially expressed in the APP model (using adjusted *P*-value < 0.01). From 1398 differentially expressed genes between Poly and control mice, 736 were present in the matched Holtman *et al.*^40^ data set and 248 of these were also differentially expressed in the APP model (again using adjusted *P*-value <0.01). The corresponding Fisher’s exact yields a *P*-value of 3.8e^−34^ and thus confirms a strong overlap of differentially regulated processes between the two models. PU.1 target genes were retrieved from Satoh *et al.*^41^ As a background control, we selected the same amount of genes that are not targeted by PU.1 but have a similar expression distribution in control samples.

### Data accessibility

Deep sequencing data are deposited on Sequence Read Archive (BioProject: PRJNA341344 accession ids: SRR4244948, SRR4244949, SRR4244950, SRR4244951, SRR4244952, SRR4244953) and will be released on publication.

### Immunohistochemistry and confocal microscopy

The detailed staining procedure was described elsewhere.^23^ For staining of the ionized calcium-binding adaptor molecule 1 (Iba1) and CD18, free-floating 40 μm thick sections were incubated in 10% donkey serum and 0.13% Triton-X in tris-buffered saline solution (TBSplus). The primary antibodies were prepared in TBSplus at the following dilutions: rabbit anti-Iba1 (Wako chemicals, Neuss, Germany, product code: 019-19741) 1:400; rat anti-CD18 (Abcam, Cambridge, UK, ab119830) 1:350. The sections were incubated with the primary antibodies overnight at 4 °C. The secondary antibodies were also prepared in TBSplus at the following dilutions: donkey anti-rabbit Cy3 (Dianova, Hamburg, Germany) 1:350; donkey anti-rat Alexa Fluor 488 (Dianova). Nuclei were stained using DAPI (4',6-diamidino-2-phenylindole), 1:500. The sections were incubated with secondary antibodies at room temperature for 2 h. Confocal Z-stacks of 20 μm of thickness were taken with a Leica SPE confocal microscope (Leica, Bensheim, Germany) through the dentate gyrus of the hippocampus at 20-times magnification. Representative pictures were taken in the proximity of the dentate gyrus at 20-times magnification. The stacks were transformed into one picture using a Z-projection with maximum intensity using ImageJ software.^42^ Mean fluorescence intensity per area for each target (Iba1, CD18, DAPI) throughout the dentate gyrus was measured by ImageJ and divided by the mean fluorescent intensity per area measured for DAPI and averaged for all animals within each group. A total of two distinct hippocampal slices per animal were sampled for the analysis. The images were sampled and analyzed by a person blind to the group identity.

### Enzyme-linked immunosorbent assay

Whole protein homogenates, from the hippocampi were obtained by mechanical tissue disruption using a syringe in ice-cold phosphate-buffered saline containing protease inhibitors (complete Ultra Tablet, Roche, Basel, Switzerland). Protein concentration was evaluated by NanoDrop 8000 spectrophotometer (Thermo Scientific, Waltham, MA, USA). Complement component 4a (C4a) levels in hippocampal lysates were measured using the mouse C4a enzyme-linked immunosorbent assay (ELISA) kit (Elabscience Biotechnology, Wuhan, China), according to the manufacturer’s manual. Detection of the cytokines was performed by using the Multiplex Immunoassay ProcartaPlex (Affymetrix eBioscence, Vienna, Austria). This beads-based ELISA allows the detection of the concentration of up to 20 analytes using the Luminex xMAP (multi-analyte profiling) technology. Specifically, the cytokines pattern included tumor necrosis factor-α and interleukin-6 (IL-6; mouse High-Sensitive Assay) combined with IL-1β (Mouse Assay).

### Flow cytometry-based phagocytosis assay

**Figure 2:**
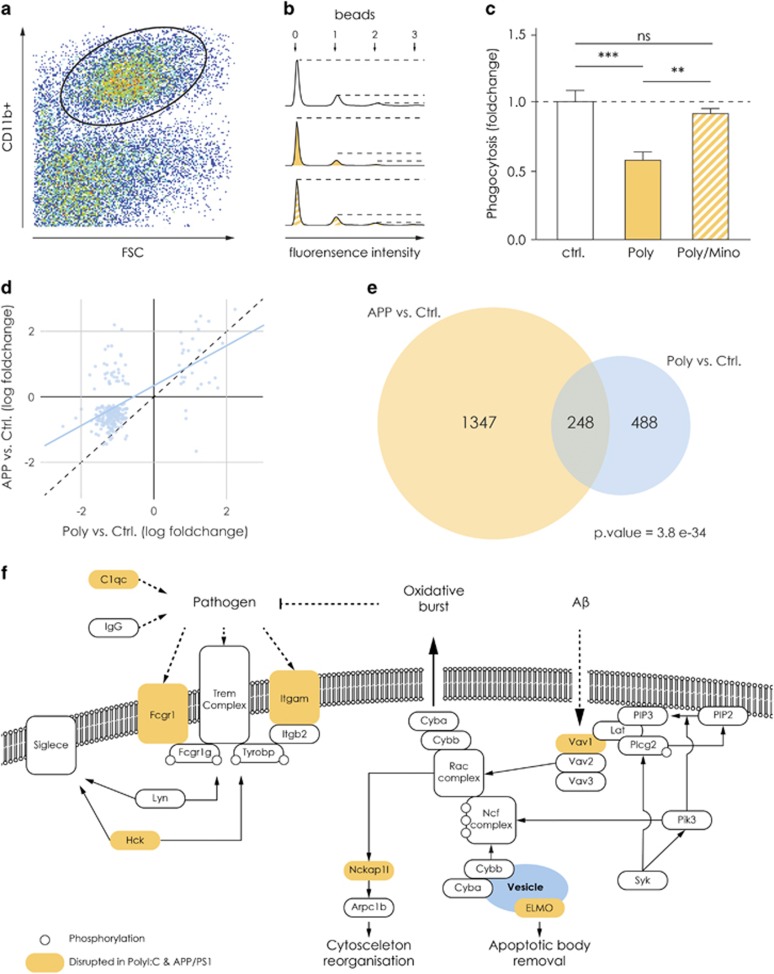
The decrease in phagocytosis and related genes are similar in the MIA and the *APP/PS1* model and rescued by minocycline treatment. (**a**) Representative image of the gating strategy used for the FACS analysis of microglia that phagocytosed fluorescent latex beads within 30 min. (**b**) Representative histograms from the three experimental groups. The peaks represent the number of microglia that phagocytosed one, two or three beads. (**c**) Graph showing the phagocytic index, which represents the total numbers of cells that phagocytosed and the number of beads taken up by a given cell. As the graph indicates, hippocampal microglia have a decreased phagocytic index in Poly(I:C) animals (*n*=12 mice) as compared with controls (*n*=17 mice). Microglial cells isolated from hippocampi of Poly(I:C) mice treated with minocycline showed a normal phagocytosis, comparable to control animals and significantly higher as compared with untreated Poly(I:C) mice (*n*=11 mice). (**d**) Scatter plot showing the comparison of the differential expression in *APP/PS1* and Poly(I:C) mice. The *x* axis represents the log2 fold changes between Poly(I:C) and control mice, *y* axis represent the log2 fold changes between *APP/PS1* and control mice. The differentially expressed genes in these two animal models correlate significantly (Pearson’s correlation=0.59). (**e**) The Venn diagram shows the overlap of significantly differentially expressed genes between *APP/PS1* versus control and Poly(I:C) versus control (for both data sets adjusted *P*-value <0.01). We found 248 genes to be significantly deregulated in both animal models as compared with their respective controls. *P*-value for the overlap was calculated with Fisher's exact test (see the 'Materials and methods' section). (**f**) Schematic representation of the pathways involved in microglial phagocytosis on different stimuli. Orange represents examples of genes found to be deregulated in microglial from both the *APP/PS1* and Poly(I:C) model. **P<0.001, ***P<0.0001. APP, *APP/SP1* mice; ctr, controls; FACS, fluorescence-activated cell sorting; MIA, maternal immune activation; NS, not significant; Poly, Poly(I:C) mice.

Microglia were isolated from the hippocampi and purified via MACS as described above. After isolation, they were resuspended in Dulbecco's Modified Eagle Medium (GIBCO, Invitrogen) supplemented with 10% fetal calf serum, 2 mm l-glutamine, 37 °C. The cells were then seeded onto primary cultured neonatal astrocytes overnight. Fluoresbrite Carboxylate Micropheres (BrightBlue, 4.5 μm) were coated with 5% fetal calf serum for 30 min at room temperature, 1000 r.p.m. The beads were resuspended in Hank's balanced salt solution at a final concentration of 2 × 10^6^ beads per ml. Microglia-astrocyte co-cultures were washed once with Hank's balanced salt solution before 1 ml bead solution was applied. The cells were incubated with the beads for 30 min at 37 °C. Afterwards, they were collected and washed in fluorescence-activated cell sorting buffer. The cells were stained with: CD11b, 1:50 (eBioscience, San Diego, CA, USA) for 15 min at 4 °C. After staining, the cells were washed in fluorescence-activated cell sorting buffer and pulled down at 500 g for 5 min. The cells were resuspended in a propidium iodide solution (1:200 in fluorescence-activated cell sorting buffer) to stain dead cells. The stained cells were transferred to a BD LSRFortessa Flow cytometer (BD Bioscience, Heidelberg, Germany). The phagocytic index as shown in Figure 2c implements the total number of cells that phagocytosed and the number of beads taken up by a given cell. The data were analyzed using FlowJo v10 software (Tree Star, Ashland, OR, USA).

### *In vitro* autoradiography

For the synthesis of [^18^F]GE180, [^18^F]fluoride was produced at a Scanditronix MC35 cyclotron via the nuclear reaction ^18^O(p,n)^18^F by proton bombardment of a ^18^O-water target, transferred to a shielded hotcell by a stream of argon and trapped on a QMA SepPak SPE cartridge (Waters, Eschborn, Germany, preconditioned with potassium bicarbonate). The [^18^F]fluoride was eluted by Kryptofix 2.2.2 (4 mg, 10 μmol) and potassium bicarbonate (1 mg, 10 μmol) in a mixture of water (0.5 ml) and acetonitrile (0.5 ml) into a sealed reaction vessel. This reaction mixture was dried under a stream of argon at 100 °C. Subsequently, without prior cooling of the mixture, the mesylate precursor (GE Healthcare; 0.5–1.0 mg) dissolved in 1 ml acetonitrile (absolute) was added, followed by 10 min of heating at 100 °C. After brief cooling, the mixture was diluted by 3.5 ml water and injected into the high-performance liquid chromatography system (Hichrom ACE 5 C18 columns, 5 μm, 100 × 10 mm) for purification. The product fraction was diluted with water (20 ml) and passed over a tC18 SepPak SPE cartridge (Waters, preconditioned with ethanol and water). After washing the cartridge with water (3 ml), the product was eluted with ethanol (0.5–1.0 ml) and formulated by further dilution with saline.

Sagittal brain sections (16 μm thick) from mice in control, Poly(I:C) and minocycline-treated Poly(I:C) animals were obtained by cryosectioning, mounted on microscopic glass slides and stored at −80 °C until the day of the experiment. The frozen brain sections were dried at room temperature in a stream of cold air, preincubated with 50 mm Tris-HCl, pH 7.4/21 °C at room temperature for 15 min and dried again before incubation with [^18^F]GE180 (0.17 MBq ml^−1^ 50 mm Tris-HCl, pH 7.4/21 °C, containing 120 mm NaCl, 5 mm KCl, 2 mm MgCl_2_, 1 mm CaCl_2_; 2.5 nm at the start of the incubation) at room temperature for 60 min. Nonspecific binding of [^18^F]GE180 was determined by co-incubation with both 20 μm GE180 and 20 μm PK11195. After incubation, the sections were washed two times for 2 min with ice-cold 50 mm Tris-HCl, pH 7.4/4 °C and rinsed in ice-cold distilled water to remove the buffer salts. The slides were dried in a stream of cold air at room temperature and exposed to ^18^F-sensitive imaging plates (BAS 2025, Fujifilm, Tokyo, Japan). The imaging plates were scanned in a CR 35 Bio (Dürr NDT, Bietigheim-Bissingen, Germany) scanner with a pixel size of 12.5 μm × 12.5 μm. Binding of [^18^F]GE180 was quantified by measuring the optical density in hand-drawn region of interest (the hippocampus) on the respective autoradiogram. The images were analyzed using Aida Image Analyzer v. 4.27 (raytest, Elysia Germany, Straubenhardt, Germany). Specific binding was calculated as total binding minus nonspecific binding for the same region in the adjacent sections. The analysis of the radioligand-binding potential was performed by a person blind to the experimental groups.

### Statistical analysis

To assess statistical differences, we used Prism 5 for Windows (Graphpad Software, La Jolla, CA, USA). For the PPI tests, we performed a two-way repeated-measures analysis of variance (ANOVA) to test for the effect of phenotype (Poly(I:C)) and treatment (minocycline) across the whole session followed by Bonferroni *post hoc* test. For the analysis of the remaining behavioral tests, the phagocytosis assay, the radioligand-binding assay, ELISA and immunohistochemically data a one-way ANOVA (when the data showed a normal distribution) followed by either Bonferroni *post hoc* test or Newman–Keuls *post hoc* test were performed. The variance between the groups was estimated through Bartlett's test for equal variances whenever the sample size was equal or bigger than 7. For smaller sample size, estimation was given based on the number of animals and standard deviation.

## Results

### Minocycline treatment rescues correlates of positive, negative and cognitive symptoms in adult Poly(I:C) animals

In the first set of experiments, we explored the effect of a chronic minocycline treatment on mouse behavioral traits similar to positive, negative and cognitive symptoms displayed by human schizophrenic patients. We used 16 Poly(I:C) animals and 14 controls for behavioral testing. Sensorimotor gating can be measured via the PPI of the startle reflex test in both humans and rodents.^14, 43^ Out of the 16 Poly(I:C) mice, 7 showed a robust deficit in sensorimotor gating (at 69, 73 and 81 dB prepulses; two-way ANOVA, F(2,66)=48.44, *P*<0.0001, Bonferroni's *post hoc* test, Poly versus controls, *P*<0.0001 for all three prepulses, Figures 1b and c). After 5 weeks of minocycline treatment (3 mg kg^−1^ per day), the same mice displayed a significant rescue in sensorimotor gating (*n*=7 Poly(I:C) mice before and after minocycline treatment and 14 controls, Bonferroni *post hoc* test Poly/Mino versus Poly, *P*<0.001 for the response to 69 dB prepulse, *P*<0.0001 for the response to 73 dB prepulse and *P*<0.001 for the response to 81 dB prepulse, Figures 1b and c). There was no difference in the response to the pulse of 120 dB alone across all tested groups and cohorts (Supplementary Figure 2). For the cognitive symptoms domain, we used the novel object recognition test for working memory.^44^ Poly(I:C) mice displayed working memory deficits in the novel object recognition test (Figures 1d and e), where their percentage of exploration of the new object over the total exploration of the objects (42%±8.8) was significantly lower compared with controls (81%±4.3). Minocycline treatment significantly improved their ability to discriminate a novel object from a familiar one (69%±4.2, *n*=16 Poly(I:C) mice before and after minocycline treatment and 14 controls, one-way ANOVA, F(2,36)=10.2, *P*=0.0003, Bonferroni *post hoc* test Poly versus control *P*<0.0001 and Poly versus Poly/Mino *P*<0.05). For readout of a negative-like symptom, we assessed social behavior. Poly(I:C) mice displayed deficits in social behavior (Figures 1f and g), shown as a significantly decreased percentage of time making contact with a stranger mouse (58%±3.8) as compared with controls (72%±3.8). After minocycline treatment, the percentage of time the mice spent with the stranger mouse normalized to control levels (70%±2.1, *n*=16 Poly(I:C) mice before and after minocycline treatment and 14 controls, one-way ANOVA, F(2,43)=7.41, *P*=0.0017, Bonferroni *post hoc* test Poly versus control *P*<0.05 and Poly versus Poly/Mino *P*<0.001). These results show similarities to the findings in human clinical trials of minocycline treatment alleviating negative, positive and cognitive symptoms in patients with schizophrenia.^16, 17, 18, 20, 21^ As deficits in rodent PPI reliably suggest a behavioral phenotype associated with schizophrenia in Poly(I:C) mice, we used this test to select only mice with a PPI deficit for all further experiments within this study. Supplementary Figures 1a–d show the final selection of the Poly(I:C) cohorts after minocycline treatment used for every experiment performed in this study. Supplementary Figures 3a–e show the single data points of the percentage of PPI for the initial selection for Poly(I:C) versus control mice before minocycline treatment.

### Hippocampal microglial cells from Poly(I:C) mice show an altered transcriptome signature that is rescued by minocycline treatment

**Figure 3:**
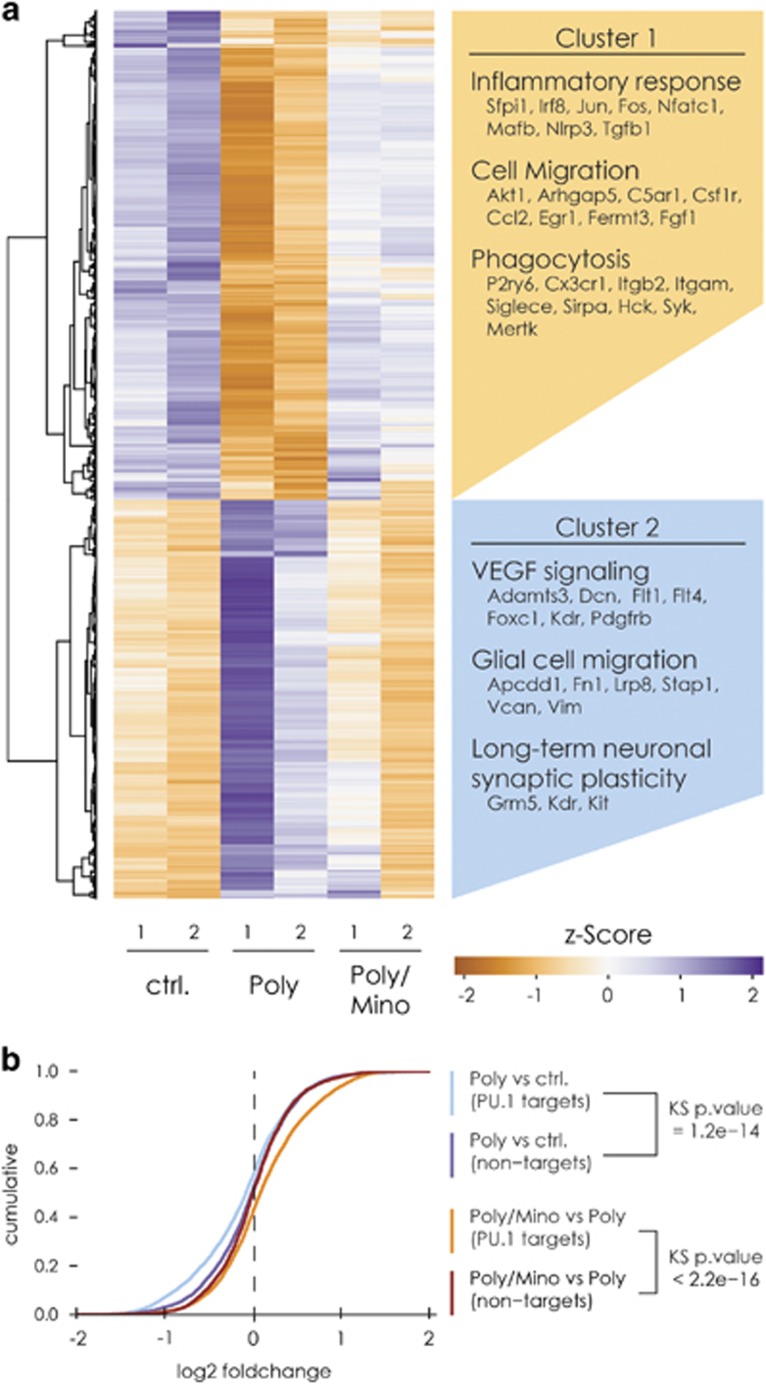
Hippocampal microglial cells from Poly(I:C) mice show a profoundly altered transcriptome signature. (**a**) Hierarchical clustering of the read counts of the differentially expressed genes between Poly(I:C) and control mice (adjusted *P*-value <0.01). The genes in cluster 1 were significantly downregulated in microglia from Poly(I:C) mice as compared with controls. The genes pertaining to cluster 2 were upregulated. Minocycline treatment brings the expression levels of genes in these clusters to a level similar to controls. Beside the clusters are displayed examples of biological processes associated with the genes within the clusters. (**b**) Empirical cumulative distribution function of the log2 fold changes of PU.1 target genes as well as non-target (expression matched) control genes. Genes targeted by PU.1 are selectively downregulated in Poly animals and minocycline treatment shifts back the expression pattern of these genes to normal levels. Kolmogorov–Smirnov two-sided test was used to calculate the *P*-values. Two biological replicates per group were used for the sequencing, where every replicate is a pool of microglia from the hippocampi of three mice.

To obtain a comprehensive overview of the pathways involved in the microglial phenotype associated with maternal immune activation during pregnancy, we performed a mRNA-seq of freshly isolated microglia from the hippocampi of adult male Poly(I:C) mice, Poly(I:C) mice treated with minocycline and control animals. A total of six animals per group were used. To obtain a substantial number of microglial cells, hippocampi were pooled together from three animals per group for a final number of two biological replicates per group. We selected Poly(I:C) animals with a significant deficit in PPI (Supplementary Figures 1a and 3b). In total, we obtained between 19.8 and 26.9 million paired-end reads per sample with 83%–91% concordant pair alignment rate (see the 'Materials and methods' section). The comparison of the replicates demonstrated a high reproducibility of the data (Supplementary Figure 4a). Notably, further comparison of the samples showed that the mRNA expression profile of the two minocycline-treated Poly(I:C) samples were similar to the control samples, while the profile of both Poly(I:C) samples were distant from the latter groups (Supplementary Figure 4b). Differential expression analysis revealed that microglia derived from the hippocampi of adult Poly(I:C) mice presents with wide-scale changes in transcriptome relative to control. We identified 1398 significantly deregulated genes (adjusted *P*-value cutoff <0.01), of which 402 were upregulated and 563 downregulated more than two fold. When comparing hippocampal microglia from Poly(I:C) mice treated with minocycline and untreated Poly(I:C) using the same cutoff as above, we found 943 deregulated genes of which 441 were upregulated and 193 downregulated more than twofold. However, we found only 21 genes to be significantly deregulated, when comparing microglial cells from minocycline-treated Poly(I:C) mice and controls already showing that minocycline normalized the Poly(I:C)-associated microglia phenotype towards the naive microglial phenotype (Figure 3a, Supplementary Table 1). To identify the deregulated biological processes in Poly(I:C) mice, we separately subjected the up- (cluster 2) and downregulated (cluster 1) genes to Gene Ontology analysis.

In cluster 1, we found significant enrichment of genes associated with processes such as inflammatory response, cell migration and phagocytosis (Figure 3a; for a complete overview of the gene ontology analysis, see Supplementary Table 2a). Of particular interest are the downregulated genes that encode for cell surface receptors associated with ‘pro-phagocytic’ signals that initiate and regulate phagocytosis. These genes include *Fcgr1, Itgav* and *P2ry6* (Figure 3a, Supplementary Table 1). Interestingly, genes also encoding microglial surface receptors that sense neuronal ‘anti-phagocytic’ signals, including *Sirpa, Siglece* and *Cx3cr1* were downregulated (Figure 3a, Supplementary Table 1). In cluster 2, we found significant enrichment of genes associated with extracellular matrix organization, embryonic development, positive regulation of long-term neuronal plasticity and angiogenesis (Figure 3a, Supplementary Table 2a).

In cluster 1, we detected a significant deregulation of *Spi1*, *Irf8* and *Jun* (Supplementary Table 1) that we validated via quantitative PCR comparing their relative expression to transcripts per million values obtained from the mRNA-seq (Supplementary Figures 4c and d). Importantly, *Spi1* and *Irf8* transcription factors may act as master switches responsible for such a drastic change in the microglial phenotype as they are crucial for microglial development and maintenance.^41, 45, 46^ Adult minocycline treatment restored the mRNA levels of these two transcription factors. In particular, *Spi1*, which encodes the transcription factor PU.1, may control around 5000 genes in microglia.^41^ We therefore compared the differential expression of previously published PU.1 target genes^41^ to expression-matched controls. This comparison demonstrated that PU.1 target genes were significantly downregulated in Poly(I:C) mice and restored on minocycline treatment (Figure 3b). We therefore hypothesize that the drastic changes in microglial transcriptome may arise from the downregulation of PU.1 expression along with its target genes.

### Increased pro-inflammatory signaling in Poly(I:C) hippocampal microglia

Studies conducted so far *in vivo* and in postmortem human brains and in the Poly(I:C) model of schizophrenia reported upregulation of classical markers of microglial activation such as increased binding of radioligands to the TSPO and increased cytokine levels (reviewed in refs 14, 47). As our transcriptome analysis reveals a marked general downregulation of genes involved in processes related to immune cell activation, we analyzed whether these markers are altered and whether minocycline prevents this alteration.

**Figure 4:**
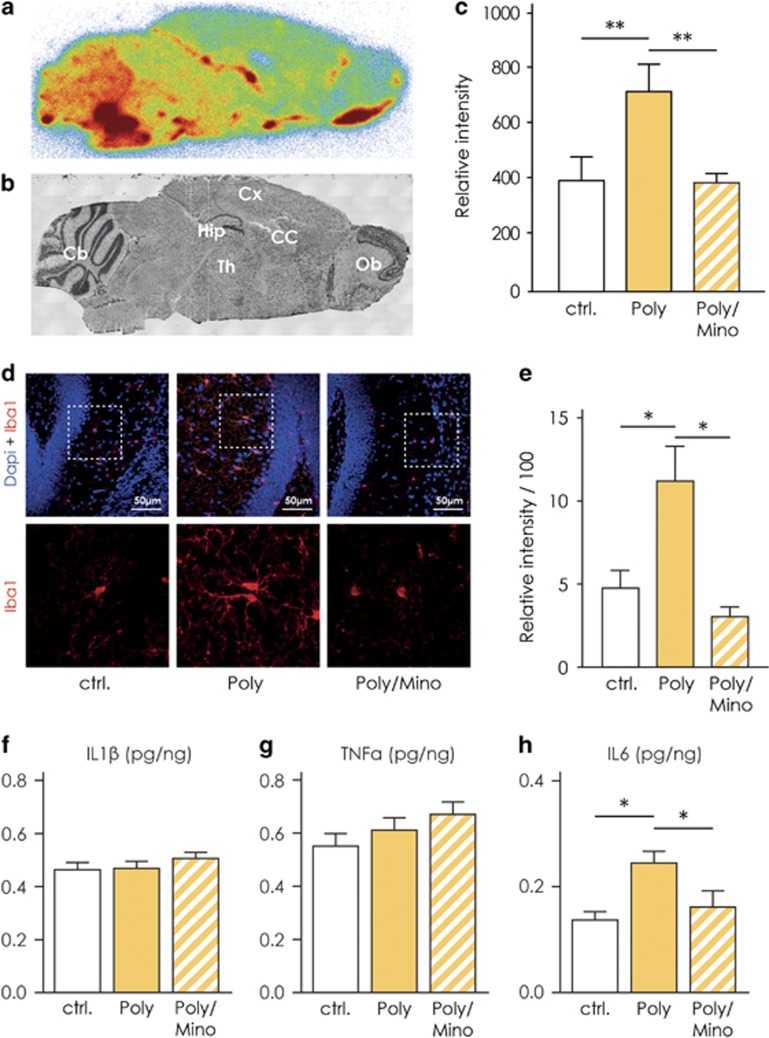
Signs of classical pro-inflammatory activation in Poly(I:C) hippocampal microglia. (**a**) Representative image of a sagittal slice (from a control animal) used for the analysis of the binding capacity with the TSPO-specific radioligand [^18^F]GE180. (**b**) Shows a representative image of a Nissl-stained sagittal slice (control animal) used to localize specific brain regions. (**c**) Shows that Poly(I:C) mice exhibit an increased binding potential to the TSPO in the hippocampus (*n*=5 mice) with respect to controls (*n*=6 mice). On the other hand, slices from Poly(I:C) mice treated with minocycline display a normalized binding potential in the latter region (*n*=6 mice), significantly lower than untreated Poly(I:C) animals and comparable to controls. (**d**) Representative pictures illustrating increased Iba1 immunoreactivity in the proximity of the DG of Poly(I:C) mice as compared with controls and minocycline-treated mice. Pictures were taken at a 63-times magnification. (**e**) Iba1 immunoreactivity was increased in the dentate gyrus of the hippocampus (DG) of Poly(I:C) mice (*n*=7) as compared with controls (*n*=5). Poly(I:C) animals treated with minocycline (*n*=4) displayed a normal Iba1 immunoreactivity. (**f**–**h**) Enzyme-linked immunosorbent assay (ELISA) measurement in whole hippocampal homogenates of the pro-inflammatory cytokines interleukin-1β (IL-1β), interleukin-6 (IL-6) and tumor necrosis factor-α (TNF-α). Only IL-6 was found to be significantly increased (**h**), while the levels of the other cytokines remained unchanged (**f** and **g**; Controls, *n*= 5; Poly(I:C), *n*=5; Poly(I:C) treated with minocycline, *n*=4). Error bars represent s.e.m. in all the panels. The data from the radioligand-binding assay were analyzed by one-way analysis of variance (ANOVA) followed by Bonferroni *post hoc* test. The data from the immunoreactivity and ELISA were analyzed by one-way ANOVA followed by Newman–Keuls *post hoc* test. *P<0.05, **P<0.001. Cb, cerebellum; Cc, corpus callosum; ctrl., control animals; Ctx, cortex; Hip, hippocampus; Ob, olfactory bulbs; Poly, Poly(I:C) animals; Poly/Mino, Poly(I:C) animals treated with minocycline; Th, thalamus; TSPO, translocator protein.

To determine region-specific TSPO binding, we performed an autoradiographic study of hippocampal slices using the TSPO ligand [^18^F]GE180.^48^ We used a strategy similar to the one adopted by Kreisl *et al.*^49^ where the authors showed that schizophrenic patients display an increased binding potential to the TSPO in postmortem brain tissue relative to controls. These findings are similar to the *in vivo* results utilizing positron emission tomography scans.^50, 51, 52^ We selected male Poly(I:C) mice with PPI deficits and randomly assigned a group of these mice to minocycline treatment and investigated the radioligand [^18^F]GE180 binding capacity (Supplementary Figures 1b and 3c). In Figure 4a, we show representative picture of a slice from a control animal, used for the analysis of binding capacity along with a slice labeled with Nissl stain to outline anatomical structures (Figure 4b). Poly(I:C) mice have an increased binding potential expressed as relative intensity of the signal detected from the bound radioligand in the hippocampus as compared with control animals (Poly, *n*=5, 808±63.4 versus controls, *n*=6, 399±87, Figure 4c). Minocycline treatment normalized the radioligand [^18^F]GE180 binding potential in the hippocampus towards control levels (Poly/Mino, *n*=6, 280±35.7, Figure 4c, one-way ANOVA F(14,16)=5.64, *P*=0.016; Bonferroni's *post hoc* test Poly versus controls and Poly/Mino versus Poly, *P*<0.05).

As a second indicator of activation, we determined the immunoreactivity of Iba1.^53^ We found that Poly(I:C) animals displayed a higher Iba1 immunoreactivity expressed as relative intensity (11±2, *n*=7) as compared with controls (4.8±1, *n*=5), while minocycline-treated mice showed an Iba1 immunoreactivity comparable to controls (3.1±0.6, *n*=4, Figures 4d and e, one-way ANOVA, F(2,13)=6.41, *P*=0.01, Newman–Keuls *post hoc* test Poly versus controls and Poly versus Poly/Mino, *P*<0.05). Next, we measured by means of ELISA the  levels (pg/ng) of the following cytokines in whole hippocampal tissue from control (*n*=5), Poly(I:C) (*n*=5) and Poly(I:C)/Mino (*n*=4) mice: IL-1β, IL-6 and tumor necrosis factor-α (Figures 4f–h). We only observed a significant increase in IL-6 in Poly(I:C) hippocampal homogenates (0.25±0.022) as compared with controls (0.14±0.016) that was abolished in the Poly(I:C)/Mino group (0.16±0.031, Figure 4h, one-way ANOVA F(2,11)=6.62, *P*=0.013, Newman–Keuls *post hoc* test Poly versus controls and Poly versus Poly/Mino, *P*<0.05). The levels of IL-1β and tumor necrosis factor-α did not change between groups (Figures 4f and g). For the PPI measurement of this cohort, see Supplementary Figures 1d and 3e.

### Decreased phagocytic activity displayed by Poly(I:C) hippocampal microglia is rescued by chronic minocycline treatment

Our transcriptome analysis indicated that genes involved in phagocytosis such as *Spi1, Syk, Hck, Dock2* and *Elmo1* are downregulated (Supplementary Table 1). To determine phagocytic activity on a functional level, we studied the incorporation of fluorescent latex beads within 30 min and quantified it by fluorescence-activated cell sorting analysis. Figure 2a shows the gating strategy to determine the uptake of the beads. In Figure 2b, we show representative histograms from all three experimental groups. The peaks represent the number of microglia that phagocytosed one, two or three beads. A detailed gating strategy is presented in Supplementary Figure 5. The phagocytosis assay conducted on hippocampal microglia revealed that these cells have a decreased phagocytic index in Poly(I:C) animals (0.58±0.06, *n*=12) as compared with controls (1.0±0.08, *n*=17, Figure 2c). Microglial cells isolated from hippocampi of Poly(I:C) mice treated with minocycline showed normal phagocytosis activity, comparable to control animals and significantly higher as compared with untreated Poly(I:C) mice (0.91±0.04, *n*=11, Figure 2c, one-way ANOVA, F(2,37)=10.21, *P*=0.003, Bonferroni *post hoc* test controls versus Poly, *P*<0.0001, Poly versus Poly/Mino, *P*<0.001). For the PPI measurements of this cohort, see Supplementary Figures 1c and 3d.

### The phenotype of microglia derived from the MIA model is comparable to the phenotype of microglia derived from the *APP/PS1* Alzheimer’s model

We previously demonstrated that microglia have decreased phagocytic functions in two mouse models of Alzheimer’s disease including the *APP/PS1* mouse.^54^ Moreover, Krstic *et al.*^25^ showed that the offspring of Poly(I:C)-challenged dams are prone to develop an Alzheimer’s-like phenotype with aging, including increased levels of amyloid precursor protein and its proteolytic fragments, as well as mislocalization and hyperphosphorylation of tau in somatodendritic compartments. To test whether the transcriptome of microglia derived from Poly(I:C) mice display any similarity to the transcriptional program attained by microglia from the *APP/PS1* mice, we compared the differentially expressed genes in microglia from the Poly(I:C) mice versus control with the differentially expressed genes in microglia from the *APP/PS1* mice versus controls (published previously^40^). We detected a high correlation between the expression changes in *APP/PS1* and Poly(I:C) mice compared with controls (Pearson’s *r*=0.59, Figure 2d). We identified 248 common deregulated genes in Poly(I:C) and *APP/PS1* microglia (Figure 2e). On gene ontology analysis, we found a significant enrichment in genes associated with phagocytosis that confirms impairment of this particular function in both animal models; similar results were obtained in the functional phagocytosis assay (Supplementary Table 2b). For a better view and understanding of the deregulated genes pertaining to different phagocytic functions, we show a schematic representation of some phagocytosis-associated pathways in Figure 2f.

### Changes in the complement system in the hippocampus of Poly(I:C) mice

**Figure 5:**
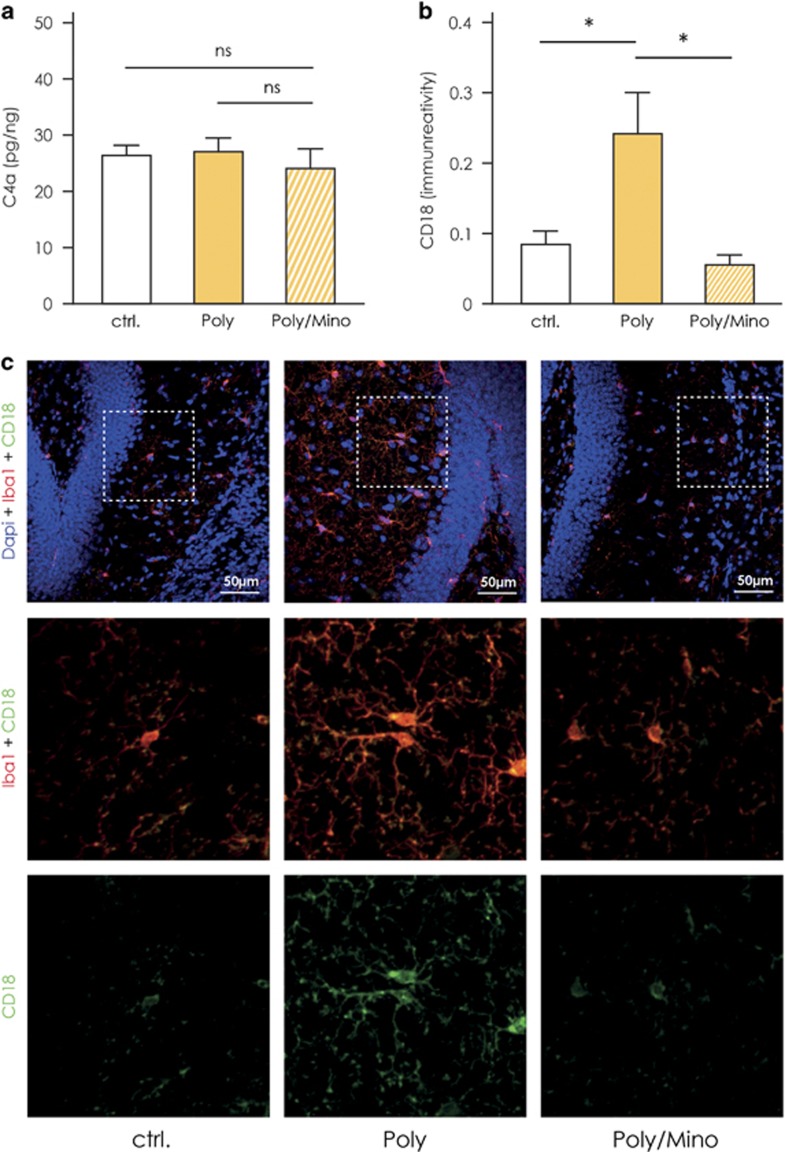
Changes in the complement system in the hippocampus of Poly(I:C) mice. (**a**) Enzyme-linked immunosorbent assay (ELISA) measurement of the complement component 4a (C4a) in whole hippocampal homogenates from Poly(I:C) (*n*=5), control (*n*=5) and minocycline-treated Poly(I:C) animals (*n*=4). No significant difference in C4a levels was detected between the groups. (**b**) Immunoreactivity for CD18 on microglia measured through the dentate gyrus (DG) of the hippocampus (DG) of Poly(I:C) (*n*=7), controls (*n*=5) and minocycline-treated Poly(I:C) mice (*n*=4). CD18 immunoreactivity in Iba1-positive cells was significantly increased in the DG of Poly(I:C) animals as compared with controls and minocycline-treated Poly(I:C) mice. (**c**) Representative pictures showing the CD18 signal co-localizing with Iba1-positive cells (microglia) and showing the increased CD18 immunoreactivity in the proximity of the DG in Poly(I:C) animals as compared with controls and minocycline-treated Poly(I:C) mice. Error bars represent s.e.m. in all the panels. The data were analyzed by one-way analysis of variance (ANOVA) followed by Newman–Keuls *post hoc* test; *P<0.05; ctrl, control animals; NS, not significant; Poly, Poly(I:C) animals; Poly/Mino, Poly(I:C) animals treated with minocycline.

The complement system consists of components crucial for proper microglial-mediated phagocytosis of synapses especially during postnatal brain development.^55^ In a recent study, Sekar *et al.*^56^ demonstrated that individual variations in the allele coding for the complement component 4 (C4) can confer high genetic risk for schizophrenia. Microglia expresses the receptor for C4, which is composed of both CD18 and CD11c.^57^ Accordingly, we measured C4a levels via ELISA on whole hippocampal homogenates and the expression of CD18 by Iba1-positive cells by fluorescent immunohistochemistry to see whether they are altered in hippocampal tissue of Poly(I:C) mice as compared with controls. We found that C4a levels (pg/ng) were unaltered in hippocampal tissue from Poly(I:C) mice (27.1±2.4, *n*=5) as compared with controls (26.4±1.8, *n*=5) and Poly(I:C) mice treated with minocycline (24.1±3.5, *n*=4, Figure 5a). On the other hand, the immunoreactivity for CD18 was significantly increased in Iba1-positive microglia residing in the dentate gyrus of the hippocampus of Poly(I:C) mice (0.24±0.059, *n*=7, Figures 5b and c) as compared with controls (0.086±0.019, *n*=5), whereas minocycline-treated Poly(I:C) animals displayed immunoreactivity comparable to baseline level (0.057±0.014, *n*=4, Figures 5b and c, one-way ANOVA F(2,13)=4.92, *P*=0.026, Newman–Keuls *post hoc* test Poly versus controls and Poly versus Poly/Mino, *P*<0.05).

## Discussion

We here demonstrate that a one-time MIA with a viral mimic during pregnancy leads to a drastic change in transcriptome and phagocytic activity of microglial cells in the hippocampus of the adult offspring. These changes were associated with schizophrenic-like behavioral abnormalities in Poly(I:C) mice. Impaired sensory-motor gating, social interaction and memory recollection in our study concur with similar findings in previous studies (reviewed in refs 14, 58). We further provide evidence of how minocycline reverses the microglial alterations on the functional and transcriptional level. Importantly, our study revealed that behavioral deficits linked with schizophrenia and changes in microglia caused by a prenatal immune challenge can be alleviated by adult minocycline treatment, indicating that the developmental changes are not irreversible. This opens the opportunity to target microglia cells in the adult as a novel therapeutic approach for schizophrenic patients. Future research should address the question as to whether the beneficial effects of minocycline on the behavioral, cellular and molecular level are long-lasting once the treatment is stopped, especially regarding microglial transcriptome signature.

Minocycline readily crosses the blood–brain barrier^59^ and is already successfully used in clinical trials and impacts human clinical data.^60^ Several studies have shown its complex effects on microglia and macrophages, which depend on the particular physiological stimulus these cells are facing.^61, 62, 63^ Although we found that minocycline reversed the MIA-induced phenotype of microglia, we are aware that it is not acting only on microglia. Its neuroprotective action along with its influence on oligodendrocytes and astrocytes may participate together with its action on microglial cells to the outcome of minocycline treatment (reviewed in ref. 64). It is also feasible that antibiotic actions of minocycline may influence microglial immune function in the central nervous system indirectly via the microbiome, which has been shown to modulate microglial cell physiology.^65^ The present study did not address minocycline effects on control animals. It would be relevant for future studies to screen the transcriptome signature of microglia from control animals treated with minocycline. However, in our previous study, when minocycline was administered to both Poly(I:C) animals and control animals, we did not observe any change of the behavioral, cellular or molecular measures tested.^23^

In the present study, hippocampal microglia from male Poly(I:C) mice displayed downregulation in expression of genes associated with cell activation, immune response, motility and phagocytosis. Of particular interest are the downregulated genes that encode cell surface receptors associated with ‘pro-phagocytosis’/‘anti-phagocytosis’ signals (*Fcgr1, Itgav, P2ry6, Sirpa, Siglece, Cx3cr1).* These receptors constitute the ‘sensome’ by which microglia and neurons communicate and they control the phagocytosis of neurons or synapses by microglia.^66^ Furthermore, genes such as *Syk, Mertk, Hck, Dock1* and *Elmo1* which encode proteins controlling cytoskeletal rearrangement and phagocytosis^67^ were also found to be downregulated in Poly(I:C) animals. Importantly, two transcription factors that are crucial for microglial cell identity, phenotype and microglia-specific functions, namely PU.1 (encoded by the gene *Sfpi1*) and interferon regulatory factor 8 (*Irf8*) were downregulated in our study.^41, 46, 68^ It was shown that silencing of PU.1 impairs phagocytosis in human microglial cultures.^46^ Comparing our data set with a recent Chip-seq study for PU.1,^41^ we found a major shift of the genes that are co-precipitated with PU.1 in Poly(I:C)-derived hippocampal microglia. Our *ex vivo* phagocytosis assay validated the fact that the phagocytic activity of hippocampal microglial cells in Poly(I:C) offspring is significantly decreased. We hypothesize that the disrupted sensome and phagocytic activity in hippocampal microglial cells participates in the behavioral dysfunctions observed in these mice.

The downregulation of phagocytosis is contrasted by increased expression of indicators of microglial activation, such as TSPO binding, Iba1 immunoreactivity and IL-6 levels. TSPO binding is not limited but mainly attributed to microglia in the current literature. Our finding is in line with the increasing evidence of a higher binding potential to TSPO in the brains of human schizophrenic patients^50, 51, 52^ especially in the hippocampus.^51^ However, in general, a direct link between changes in TSPO and microglial activation should be addressed with care. Studies have indeed demonstrated changes in microglial TSPO expression and binding potential in pathological conditions. Nevertheless, TSPO is expressed also by other brain cells such as ependymal and endothelial cells.^69^ In addition, we observed a stronger radioligand binding in certain brain regions such as the brain stem and olfactory bulbs (see Figure 4a). This is likely due to the fact that TSPO is a mitochondrial protein responsible for the translocation of cholesterol and, therefore, it may show different expression patterns according to the specific metabolic needs of a particular brain region at any given moment.

It is important to emphasize that CD18 and Iba1 were significantly downregulated at the mRNA level, but showed an increased immunoreactivity at the protein level. Divergent mRNA and protein kinetics may depend on the relative half-life of a given mRNA versus its protein, which may not correlate.^70^ Our data show that the kinetic of protein levels might not correspond with the kinetic of mRNA levels, which might also contribute to the controversial findings between studies.

Previous studies utilizing the maternal immune activation model including ours focused either on microglia density or cytokine production.^23, 71, 72, 73, 74^ Although some studies including the evaluation of human postmortem tissue reported increased microglia density in defined brain areas, others did not find major changes. Similar controversial data exist for cytokine levels in the brain of patients with schizophrenia as well as in the present animal model (reviewed in ref. 3). These differences could be attributed to microglia heterogeneity with respect to region, gender or age. One recent study highlighted the importance of microglia temporal heterogeneity during development.^75^ A number of studies showed that microglia attain different transcriptional programs in distinct brain regions^76, 77, 78^ or differ between male and female brains.^79^ Thus, when comparing data, it is important to consider the developmental time point, the brain region and gender as these factors might attribute to the reported differences between the above-mentioned studies.

Microglial cell polarization is a complex matter, as it has been demonstrated in Alzheimer’s disease where microglia can co-express classical markers of activation concomitant with markers of opposite, non-classically activated phenotypes.^80^ This is in line with the microglia phenotype we describe in the current study: an increase in IL-6, TSPO binding and Iba1 reactivity is accompanied by unchanged levels of tumor necrosis factor-α and IL-1β and impairment in phagocytosis. We have previously reported impairment in phagocytosis of microglia associated with plaques in the *APP/PS1* model for Alzheimer’s disease using a bead-based phagocytosis assay similar what was used in the current study.^54^ It was also shown that immunotherapeutic amyloid clearance was less effective and microglia response altered when phagocytosis was impaired.^81, 82^ Thus, downregulation of functional microglial phagocytosis is a common feature in Alzheimer’s disease and MIA models. When comparing the microglial transcriptome changes in our model (Poly(I:C) versus control) with the changes observed in the *APP/PS1* mice (*APP/PS1* versus control),^40^ we see a strong overlap in genes of the phagocytosis pathway. Vav1 and Elmo have been shown to be crucial for fibrillary β-amyloid-stimulated oxidative burst and phagocytosis.^83^ Considering this overlap, especially in terms of phagocytic functions and the complex phenotype attained by Poly(I:C) microglia, our data support the hypothesis that early immune challenge can lead to an Alzheimer’s-like pathology as proposed by Krstic and colleagues.^25, 26^ Data-driven analyses of brain structural variation by magnetic resonance imaging revealed a network of brain regions that spatially recapitulates the pattern of brain abnormalities observed in both schizophrenia and Alzheimer’s disease. The authors of this study suggest that the common spatial pattern of abnormalities observed in these two disorders might be influenced by the timing of their separate and distinct pathological processes in disrupting cerebral development and aging, respectively.^84^

With respect to the complement-linked phagocytic activity, it has been shown that complement component C3, complement receptor type 3 and C1q contribute to the early and excessive synaptic loss through microglia-mediated phagocytosis in a model of Alzheimer’s disease.^85^ In a recent study, Sekar *et al.*^56^ found an association between schizophrenia and variations in the major histocompatibility complex locus on alleles affecting the expression of the complement component 4 (C4) particularly in the hippocampus of schizophrenic patients.^56^ The main function of C4 is to activate the complement component 3 (C3) for proper synaptic elimination during development, and the authors suggest this as a mechanism by which schizophrenic individuals exhibit decreased number of synapses in diverse brain regions in postmortem studies.^56^ In our mouse model, we did not detect changes of C4a protein in the hippocampal homogenates between the groups, which is not surprising as the MIA mice do not harbor a genetic C4 mutation like the human subjects. However, changes in CD18 (part of the receptor for C3 and C4) indicates that prenatal insult perturbs the complement system also in the absence of genetic background, possibly participating to the development of a defect brain micro-circuitry.

We propose that an immune challenge during prenatal development causes changes in microglial function in adulthood that shares common features with changes in microglial function in neurodegenerative diseases such as Alzheimer’s disease. Early-life events such as MIA might contribute to the neurogenic reserve by setting the basis on how an individual might withstand secondary challenges later in life.^86^ Lahiri *et al.*^28^ propose a 'Latent Early-life Associated Regulation' model, positing latent changes in the expression of specific genes initially primed at the developmental stage of life. In this model, environmental agents epigenetically disturb gene regulation in a long-term manner, beginning at early developmental stages, but these perturbations might not have pathological results until significantly later in life.^28^ Our data provide evidence for this hypothesis on the level of microglia functionality. By identifying common denominators and traits in microglia derived from mouse models of neurodegenerative and psychiatric disorders, we here highlight the importance of the brain’s immune system as a target for add-on therapeutic interventions, already starting before the onset of symptoms.
