# Supplementary material for: Maternal immune activation results in complex microglial transcriptome signature in the adult offspring that is reversed by minocycline treatment
Source: Transl Psychiatry. 2017 May 9;7(5):e1120–. doi: 10.1038/tp.2017.80 (PMC5534948; doi:10.1038/tp.2017.80)
Supplement: Supplementary Material 1 [file tp201780x3.pdf]

Sfpi1	5' –> 3'	
Forward	ATGTTACAGGCGTGCAAAATGG	
Reverse	TGATCGCTATGGCTTTCTCCA	

Irf8	
Forward	CGGGGCTGATCTGGGAAAAT
Reverse	CACAGCGTAACCTCGTCTTC
Tgf1b	
Forward	AGGGCTACCATGCCAACTTC
Reverse	TGGTTGTAGAGGGCAAGGAC
Jun	
Forward	CCTTCTACGACGATGCCCTC
Reverse	GGTTCAAGGTCATGCTCTGTTT
Ptgs2	
Forward	ATCCTTGCTGTTCCAATCCA
Reverse	ATCCAGTCCGGGTACAGTCA
Vim	
Forward	TTCTCTGGCACGTCTTGACC
Reverse	GCTTGGAAACGTCCACATCG
Cx3cr1	
Forward	CCTGTTATTTGGGCGACATT
Reverse	ACCAGACCGAACGTGAAGAC
