# Supplementary figures and images for: Maternal immune activation results in complex microglial transcriptome signature in the adult offspring that is reversed by minocycline treatment
Source: Transl Psychiatry. 2017 May 9;7(5):e1120–. doi: 10.1038/tp.2017.80 (PMC5534948; doi:10.1038/tp.2017.80)

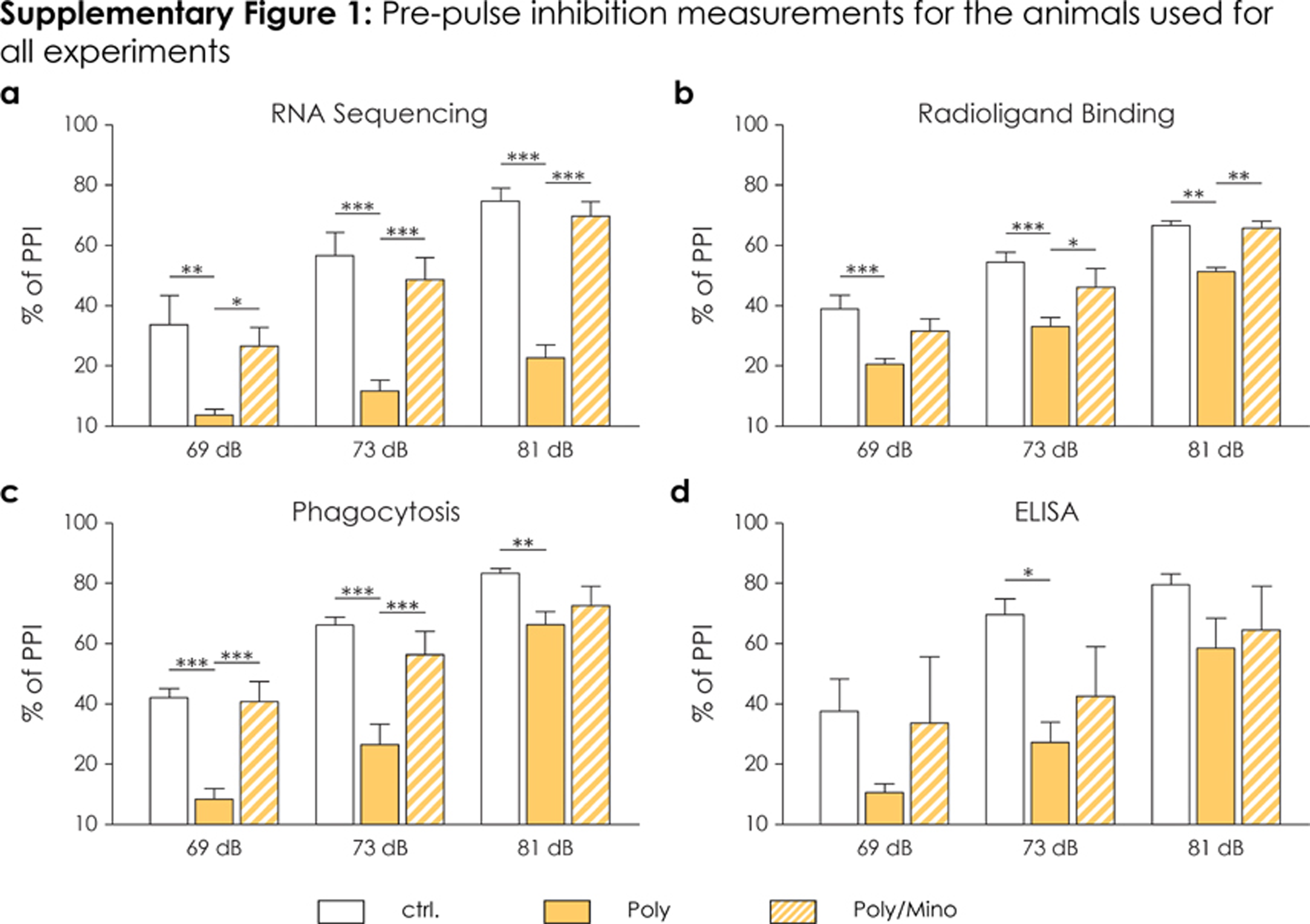

Supplement: Supplementary Figure 1 [file tp201780x4.tif]

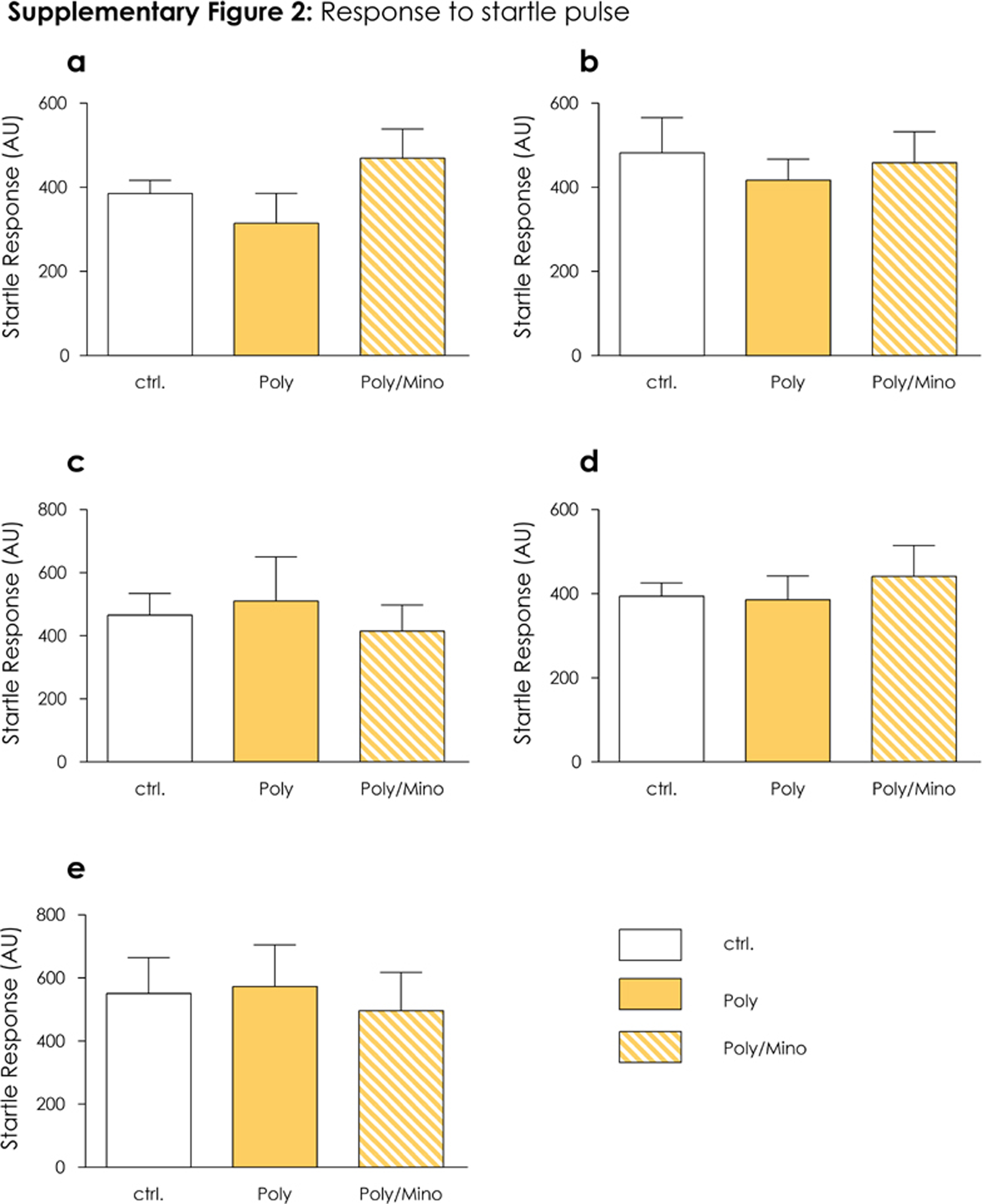

Supplement: Supplementary Figure 2 [file tp201780x5.tif]

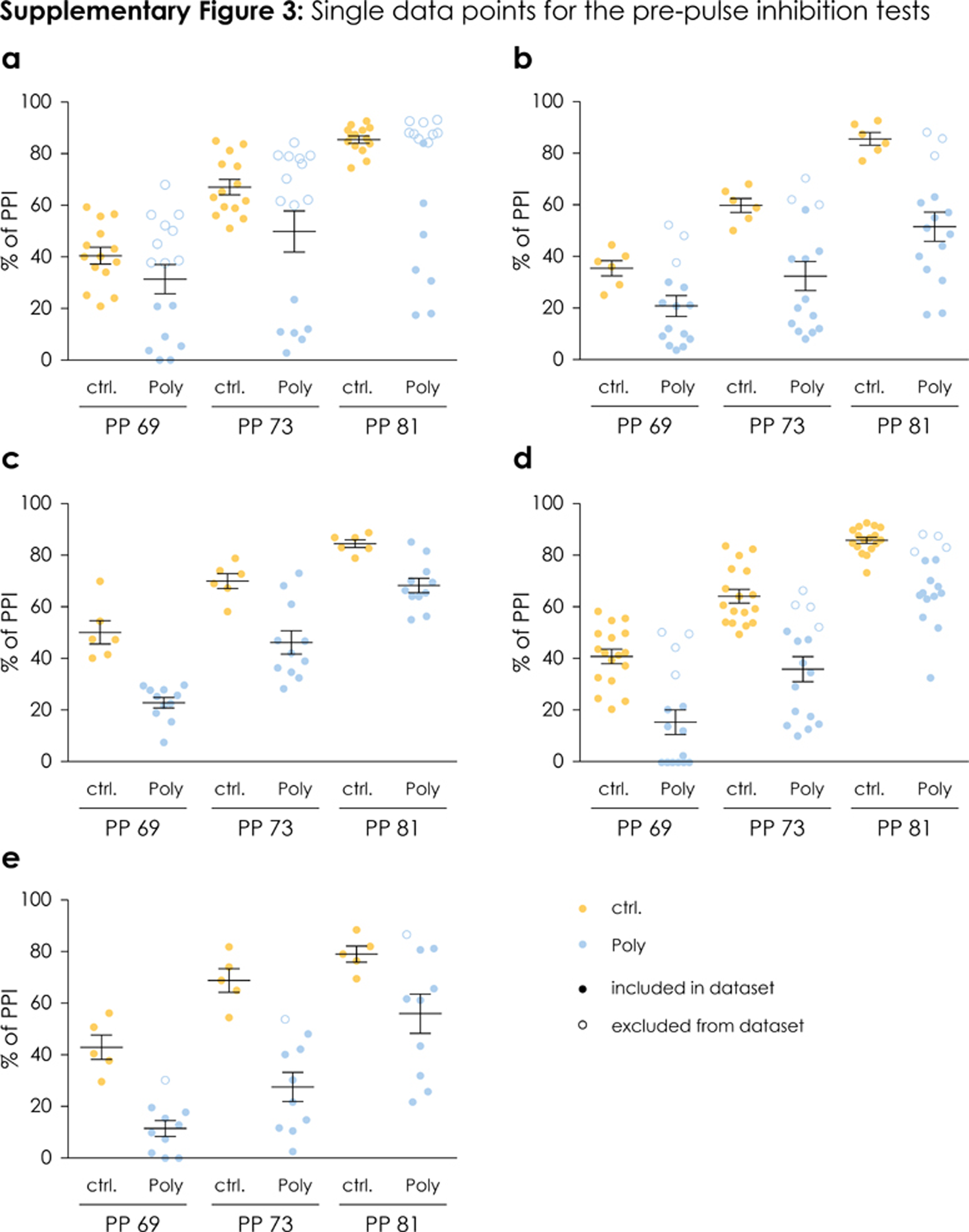

Supplement: Supplementary Figure 3 [file tp201780x6.tif]

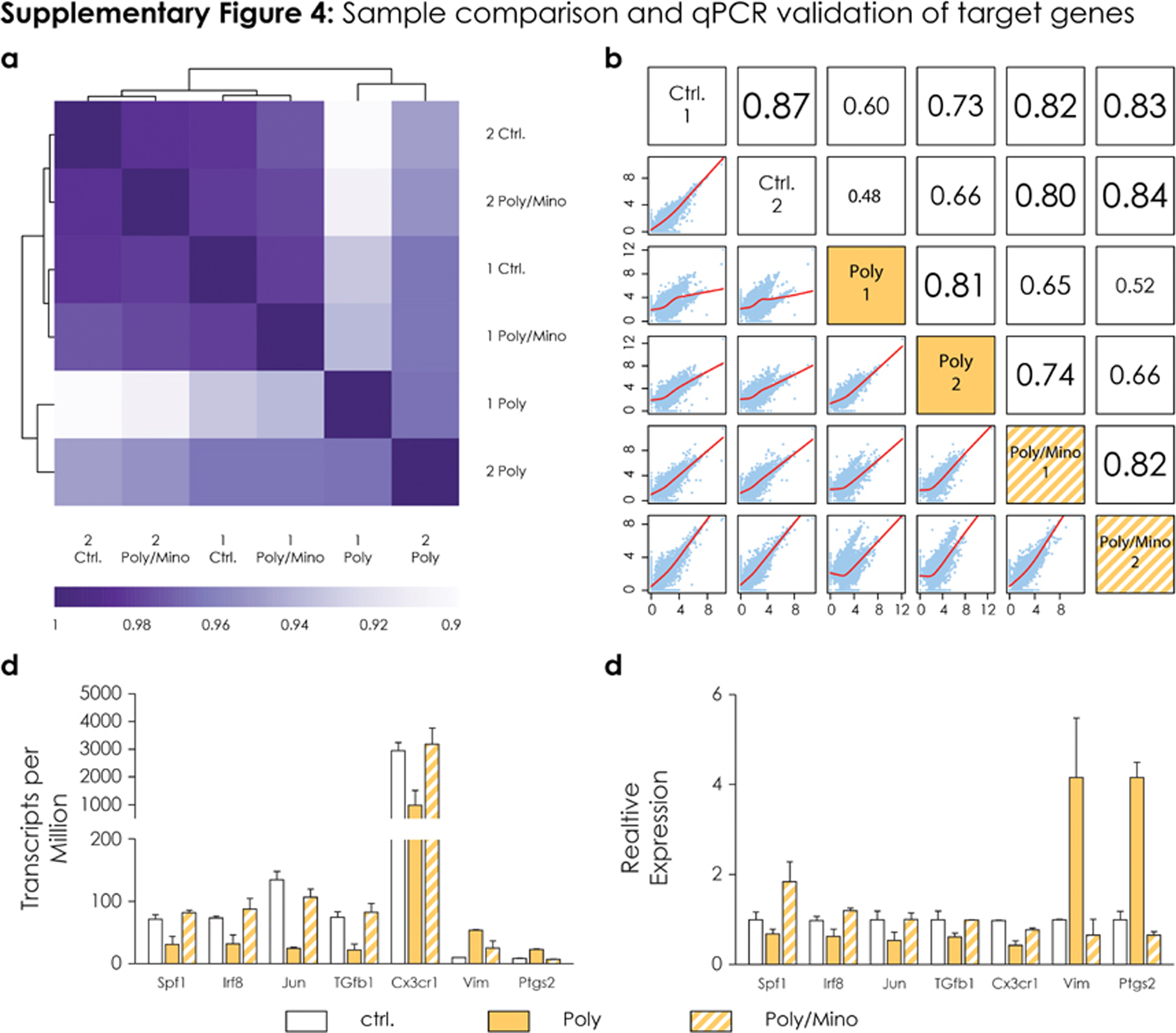

Supplement: Supplementary Figure 4 [file tp201780x7.tif]

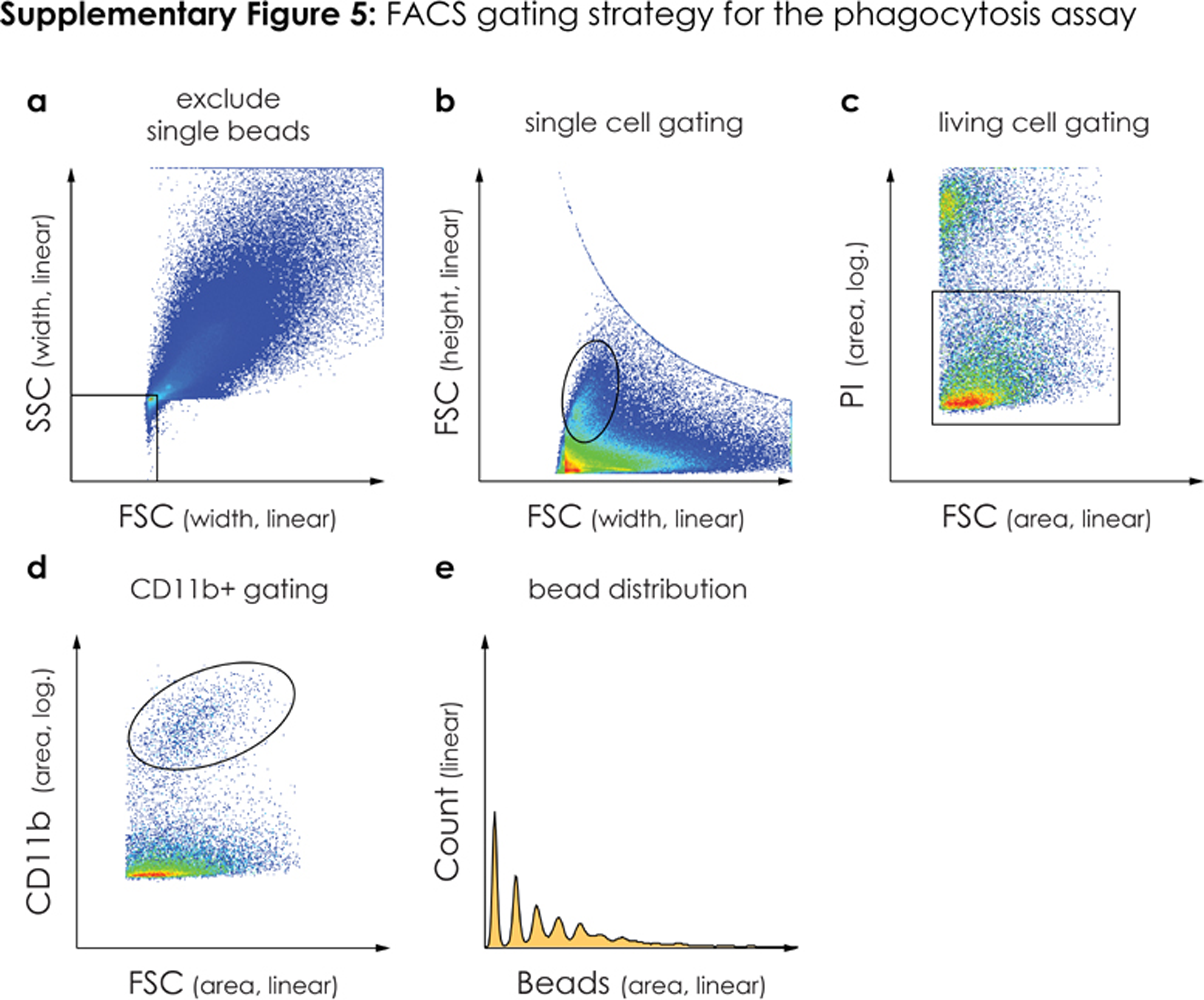

Supplement: Supplementary Figure 5 [file tp201780x8.tif]
